# Sp1‐mediated ectopic expression of T‐cell lymphoma invasion and metastasis 2 in hepatocellular carcinoma

**DOI:** 10.1002/cam4.611

**Published:** 2016-01-14

**Authors:** Wei‐Hsuan Yen, Wu‐Sian Ke, Jan‐Jong Hung, Tsung‐Ming Chen, Jia‐Shing Chen, H. S. Sun

**Affiliations:** ^1^ Institute of Molecular MedicineCollege of MedicineNational Cheng Kung UniversityTainan70101Taiwan; ^2^ Institute of Bioinformatics and Biosignal TransductionCollege of Bioscience and BiotechnologyNational Cheng Kung UniversityTainan70101Taiwan; ^3^ Department of PhysiologyCollege of MedicineNational Cheng Kung UniversityTainan70101Taiwan

**Keywords:** Ectopic activation, GC box, Sp1, TATA‐less gene, TIAM2S

## Abstract

T‐cell lymphoma invasion and metastasis 2 (*TIAM2*) is a neuron‐specific protein that has been found ectopically expressed in hepatocellular carcinoma (HCC). Results from clinical specimens and cellular and animal models have shown that the short form of *TIAM2* (*TIAM2S*) functions as an oncogene in the tumorigenesis of liver cancer. However, the regulation of *TIAM2S* ectopic expression in HCC cells remains largely unknown. This study aimed to identify the mechanism underlying the ectopic expression of *TIAM2S* in liver cancer cells. In this report, we provide evidence illustrating that Sp1 binds directly to the GC box located in the *TIAM2S* core promoter. We further demonstrated that overexpression of Sp1 in HepaRG cells promotes endogenous *TIAM2S* mRNA and protein expressions, and knockdown of Sp1 in 2 HCC cell lines, HepG2 and PLC/PRF/5, led to a substantial reduction in *TIAM2S* mRNA and protein in these cells. Of 60 paired HCC samples, 70% showed a significant increase (from 1.1‐ to 3.6‐fold) in Sp1 protein expression in the tumor cells. The elevated Sp1 expression was highly correlated with both *TIAM2S* mRNA and protein expressions in these samples. Together, these results illustrate that Sp1 positively controls *TIAM2S* transcription and that Sp1‐mediated transcriptional activation is essential for *TIAM2S* ectopic expression in liver cancer cells.

## Introduction

The T‐cell lymphoma invasion and metastasis 2 (*TIAM2*) gene is located on chromosome 6q25.2 and is the homolog of human T‐cell lymphoma invasion and metastasis 1 (*TIAM1*). Although the role of TIAM1 in neuron development and human malignancies are well characterized, the physiological and pathological functions of TIAM2 were largely unknown. Our previous study showed that TIAM2 was overexpressed in a great majority (86%) of hepatocellular carcinoma (HCC) samples and significantly associated with *TIAM1* overexpression 1. Our experiments also have demonstrated that overexpression of *TIAM2* promotes cancer cell proliferation and increases the invasiveness of HCC. These data provided the first evidence that the induced expression of endogenous *TIAM2* in liver cancer promotes epithelial‐to‐mesenchymal transition (EMT) and results in the proliferation of and invasion by liver cancer cells 1. Recently, a study by Zhao et al. 2. showed that *TIAM2* promotes cell invasion and motility in nonsmall cell lung cancer by activating *Rac1* and EMT‐associated genes. Although the mechanism remains unclear, these data demonstrate that aberrant expression of the short form of *TIAM2* (*TIAM2S*) underlies *TIAM2*‐mediated tumorigenesis.

Human *TIAM2* encodes two transcripts, the long form of *TIAM2* (*TIAM2L*) (NM_012454.3, also known as *TIAM2* variant 1) and *TIAM2S* (NM_001010927.2, also known as *TIAM2* variant 2). *TIAM2L* was predicted to encode a large protein with 1077 amino acids, whereas the deduced protein of *TIAM2S* was about approximately 626 amino acids. Although *TIAM2L* mRNA was highly expressed in many human tissues, no *TIAM2L* protein was detected in any of the tissues examined 1. In addition, the detection of the *TIAM2S* protein only in the normal human brain suggested *TIAM2S* may be a brain‐specific protein and involved in brain function. Nevertheless, *TIAM2S* was found ectopically expressed in HCC cells and the regulatory mechanism controlling *TIAM2S* expression in HCC was unknown 1.

The aberrant regulation of transcription factors (TFs) is closely related to various human diseases. For example, the androgen receptor (*AR*), a nuclear TF that regulates many gene expressions involved in the development and maintenance of the male sexual phenotype, is involved in the progression of prostate cancer when aberrantly expressed 3. Another example is specificity protein 1 (Sp1), a TF belonging to the specificity protein/Krüppel‐like factor (SP/KLF) family that regulates many cellular physiological processes such as metabolism, cell growth, differentiation, angiogenesis, and apoptosis 4. The aberrant expression of Sp1 contributes to the tumorigenesis of various types of cancer 5.

Sp1 regulates its target gene transcription activity by directly binding to GC‐rich motifs with greater affinity of many TATA‐box‐containing or TATA‐less promoters 6, 7, 8. Recent studies have shown that the transcriptional activity of Sp1 is mediated by its posttranslational modifications (PTM), which affect transcriptional activity, DNA‐binding affinity, and Sp1 protein levels 9. For example, Sp1 phosphorylation at Thr739 increases its stability by preventing interaction with Really Interesting New Gene (RING) finger protein 4 (RNF4), thus protecting Sp1 from proteasome‐dependent degradation 10. Additionally, Sp1 sumoylation at Lys16 by SUMO‐1 facilitates Sp1 proteolytic processing, which consequently alters subcellular location and leads to ubiquitin‐dependent degradation. Sp1 sumoylation levels are reduced in tumorous cervical tissues, which suggests that Sp1 accumulation correlates with sumoylation inhibition during tumorigenesis 11. Furthermore, Sp1 deacetylation at Lys703 increases its transcriptional activity and recruitment of p300 and increased target gene expression 12. The O‐linked glycosylation (O‐GlcNAc) of the activation domain prevents binding with *TAF*II110, thus repressing Sp1‐mediated transcription 13.

Although Sp1 is a common TF, Sp1‐dependent transcription is highly regulated and heavily involved in the development of various cancers, including lung 14 and gastric cancer 15. In both clinical specimens and cancer models, previous studies have revealed that Sp1 levels correlate with stage, invasive potential, metastasis, and even patient survival 5. Furthermore, previous studies have indicated that Sp1 might extend tumor growth and metastasis through the overexpression of many Sp1 target downstream genes, including mesenchymal factors, genes that promote cell proliferation, and oncogenes. For example, Sp1 can induce TGF‐*β*‐mediated EMT through the activation of Snail expression 16 and also cooperate with activated Smad complexes to express EMT‐associated marker genes 17. The results of these studies support the proposal that elevated Sp1 expression contributes to cancer development and progression and represents a potential risk of poor prognosis. Because a GC box was predicted in the *TIAM2S* promoter region, we hypothesized that Sp1 may play a role in controlling *TIAM2S* expression. Therefore, the objective of this study was to investigate whether Sp1‐mediated transcriptional activation contributes to the ectopic expression of *TIAM2S* in liver cancer cells.

## Materials and Methods

### Specimens and cell lines

We used 68 paired HCC samples (tumor and matched nontumor) to investigate the correlation of expressions between Sp1 and *TIAM2S* in HCCs. All tissue samples were freshly frozen at −80°C until further analysis. The detailed information of both HCC patients and cell lines was provided in a previous study 1.

Two HCC cell lines (HepG2 and PLC/PRF/5) and one terminally differentiated hepatic cell line that retained many characteristics of primary human hepatocytes (HepaRG) were used in this study. In addition, one neuroblastoma cell line (IMR32) that expressed endogenous *TIAM2S* and one pluripotent cell line (NTER‐2c1.D1; NT2/D1) that expressed both endogenous Sp1 and *TIAM2S* proteins were also used. All cell lines were maintained in a 37°C incubator with 5% CO_2_, in accordance with the protocol suggested by the American Type Culture Collection (ATCC).

### Computational prediction, promoter constructs, and luciferase reporter assays

Computational tools, including the PROMO (http://alggen.lsi.upc.es/), Transcription Element Search System (TESS; http://www.cbil.upenn.edu/cgi-bin/tess/tess), Eukaryotic Promoter Database (EPD, http://www.epd.isb-sib.ch/), and Searching Transcription Factor Binding Sites (TFSEARCH; http://www.cbrc.jp/research/db/TFSEARCH.html), were used to predict the potential TF‐binding sites and promoter on the human *TIAM2S* sequences (upstream from the *TIAM2S* mRNA; NCBI reference sequence NM_001010927.2).

To clone the *TIAM2S* promoter regions for further analysis, primers were designed (Table S1) based on the human *TIAM2S* sequences (NCBI accession number: Z11168.1). Using either sequence‐containing or primer‐anchored restriction enzyme cutting sites, fragments representing series deletions of *TIAM2S* sequences were cloned into the pGL3‐basic vector (Promega, Madison, WI).

Approximately 2 × 10^5^ cells from different cell lines were transfected with various reporter constructs. We performed luciferase assays according to the protocol described previously 18. Firefly luciferase activity was normalized to *β*‐Galactosidase enzyme activity and presented relative to the luminescence driven by the pGL3‐promoter (relative luciferase units; RLU). In addition, the effect of Sp1 overexpression on the activity of the *TIAM2* promoter was assessed using 125‐ng *TIAM2S* promoter deletion constructs and 375‐ng pGFP‐Sp1 constructs 19 in PLC/PRF/5 cells.

### Total, nuclear, and cytosol protein preparation

For total protein preparation, approximately 6 × 10^6^ cells were lysed in a RIPA buffer (50 mmol/L tris‐HCl pH 7.5, 150 mmol/L NaCl, 0.1% SDS, 0.5% sodium deoxycholate, 1% NP‐40, 1 mmol/L PMSF, 1 mmol/L dithiothreitol, 1X protease inhibitor) for 10 min on ice, followed by centrifugation at 16,000*g* for 15 min to remove the debris.

For preparation of the nuclear and cytosol lysates, approximately 6 × 10^6^ cells washed with ice‐cold PBS and lysed in Cytosol Extract Reagent (10 mmol/L HEPES, pH 7.9, 10 mmol/L KCL, 1.5 mmol/L MgCl_2_, 0.5 mmol/L DTT, 1X protease inhibitor, 5% glycerol, 10% Triton X‐100) for 10 min on ice. After centrifugation at 16,000*g* for 15 min, the cytosolic fraction was collected from the top liquid phase; while the bottom pellet was followed up for nuclear lysate extraction. The pellets were lysed in a Nuclear Extract Reagent (20 mmol/L HEPES, pH 7.9, 450 mmol/L NaCl, 1.5 mmol/L MgCl_2_, 0.2 mmol/L EDTA, 0.5 mmol/L DTT, 1X protease inhibitor, 25% glycerol) for 40 min on ice, with stringent vortex every 10 min. This procedure was followed by centrifugation at 16,000*g* for 10 min. The supernatants were collected as nuclear extracts.

### RNA isolation and quantitative real‐time polymerase chain reaction

The total RNA from the HepG2 and PLC/PRF/5 cells was isolated using TRIzol^®^ Reagent (Ambion, Austin, TX) according to the manufacturer's protocol. Potential DNA contamination was removed using the TURBO DNA‐free^TM^ Kit (Ambion). The DNA‐free RNA samples were used for cDNA synthesis using High‐Capacity cDNA Reverse Transcription Kits (Applied Biosystems, Foster City, CA) and subjected to quantitative real‐time polymerase chain reaction (PCR) with indicating primer sets (HS99999901‐s1 for *TIAM2S* and ACTB; Applied Biosystems) in a sequence detector (ABI StepOne Plus^TM^; Applied Biosystems). The levels of *TIAM2S* mRNA in samples were measured using the 2^−△△Ct^ relative quantification method and normalized to *β*‐actin as an internal control. All measurements were performed in triplicate, and the experiments were repeated at least twice.

### Western blotting and antibodies

Proteins were fractionated using SDS‐PAGE and transferred to a polyvinylidene difluoride (PVDF) membrane (Millipore, Bedford, MA). Membranes were blocked in TBST buffer (10 mmol/L Tris‐HCl pH 7.5, 150 mmol/L NaCl, and 0.05% Tween 20) containing 5% nonfat milk for at least 1 h and incubated overnight with appropriate antibodies at 4°C. The membranes were washed four times in TBST and incubated with a horseradish peroxidase secondary antibody for 1 h. Signal detection was performed using an enhanced chemiluminescence (ECL) detection system (PerkinElmer Life Science, Waltham, MA).

The antibodies used in this study were anti‐*TIAM2* (P‐17) (1:200; Santa Cruz, Paso Robles, CA), anti‐Sp1 (1:3000; Millipore), anti‐*β*‐actin (1:2000; Abcam, Cambridge, UK), anti‐*α*‐tubulin (1:2000; Cell signaling, Danvers, MA), and anti‐pSp1‐T739 19.

### Electrophoretic mobility shift assay

To examine the formation of DNA‐protein complexes, biotinylated oligoribonucleotides representing upstream −75 to −51 sequences from the transcription start site (TSS) of *TIAM2S* (5′‐CTGCTGTGGAGGAAGAGCTTGGTGGC‐3′) were synthesized (Integrated DNA Technologies, Inc., Coralville, IA) and used for further analysis. To perform the electrophoretic mobility shift assay (EMSA), 20 *μ*g of nuclear extracts from HepG2 cells were prepared and incubated with a biotin‐labeled DNA probe according to the protocol described previously 20. For the competition assay, 2‐, 5‐, or 10‐fold of an unlabeled oligoribonucleotide probe was added to the binding reaction. The biotin‐labeled probes were then added for further incubation for 30 min at room temperature. For the supershift assays, the nuclear proteins were first incubated with specific antibodies at room temperature for 1 h, followed by the aforementioned procedure. The signal intensities were quantified using the spot density function with a gel documentation system (AlphaImager, Alpha Innotech). The reduction rate was calculated as the spot density of specific shifted band divided by the spot density of free DNA probe in each lane, then normalize to the value of lane 2 (Nuclear extract + Biotin probe).

### Chromatin immunoprecipitation assay

To confirm the DNA–protein interactions, cell lysates were prepared from approximately 4.5 × 10^6^ HepG2 cells and 5 × 10^6^ PLC/PRF/5 cells and used in ChIP assays according to the procedure described previously 21. Normal rabbit IgG replaced the Sp1 antibody as the negative control. After protein digestion by proteinase K at 50°C for 2 h, DNA was extracted using phenol/chloroform and precipitated by isopropanol. The purified DNA was subjected to PCR using a primer designed from −182 to +65 of the *TIAM2S* promoter sequences (F: 5′‐GTCCCATTGTCTCCACGTCT‐3′; R: 5′‐TAAGTCGGCTGTTG GGAGAT‐3′).

### Transient overexpression and RNA interference

In previous experiments examining Sp1 overexpression, approximately 4 × 10^5^ PLC/PRF/5 or 6 × 10^5^ HepG2 cells were infected with adenovirus‐expressing GFP‐Sp1 22 for 24 h. The overexpression of the Sp1 protein was confirmed using Western blotting. The effect of Sp1 overexpression on reporter gene activity was measured using luciferase assays. For the detection of Sp1 PTM assay, approximately 5 × 10^5^ PLC/PRF/5 cells were transfected with 1 *μ*g of each construct (pEGFP‐N1, pGFP‐Sp1, pGFP‐Sp1‐T739A, and pGFP‐Sp1‐T739D; 19 in a six‐well plate. After 24 h of transfection, cells were lysed with a RIPA buffer. Whole‐cell extracts and the total RNA of PLC/PRF/5 cells were collected for Western blotting and real‐time PCR analysis.

To knock down Sp1 expression in HepG2 or PLC/PRF/5 cells, lentiviral shRNA clones that target the *Luc* (TRCN0000231693) or *SP1* (TRCN0000020444, TRCN0000274153, TRCN0000274208) genes were purchased from the National RNAi Core Facility of Academia Sinica (http://rnai.genmed.sinica.edu.tw/). In Sp1 knockdown experiments, approximately 1.9 × 10^5^ HepG2 or PLC/PRF/5 cells/well were incubated in six‐well plates for 16 h, followed by infection with shSp1 lentiviruses (multiplicity of infection, MOI = 10) for 48 h. The knockdown efficiency was measured using Western blot analysis, and the effect of Sp1 knockdown on reporter gene activity was measured using luciferase assays. All knockdown and overexpression experiments were performed in triplicates and were independently performed at least three times.

### Statistical analysis

All experimental data were analyzed using GraphPad Prism 5.0 (GraphPad Software, Inc., San Diego, CA) and presented as the mean ± standard error of the mean (SEM). The results were further analyzed using the Student's *t* test or one‐way analysis of variance (ANOVA). The significance level for all statistical tests was 0.05.

## Results

### Defining the *TIAM2S* minimal core promoter region

**Figure 1:**
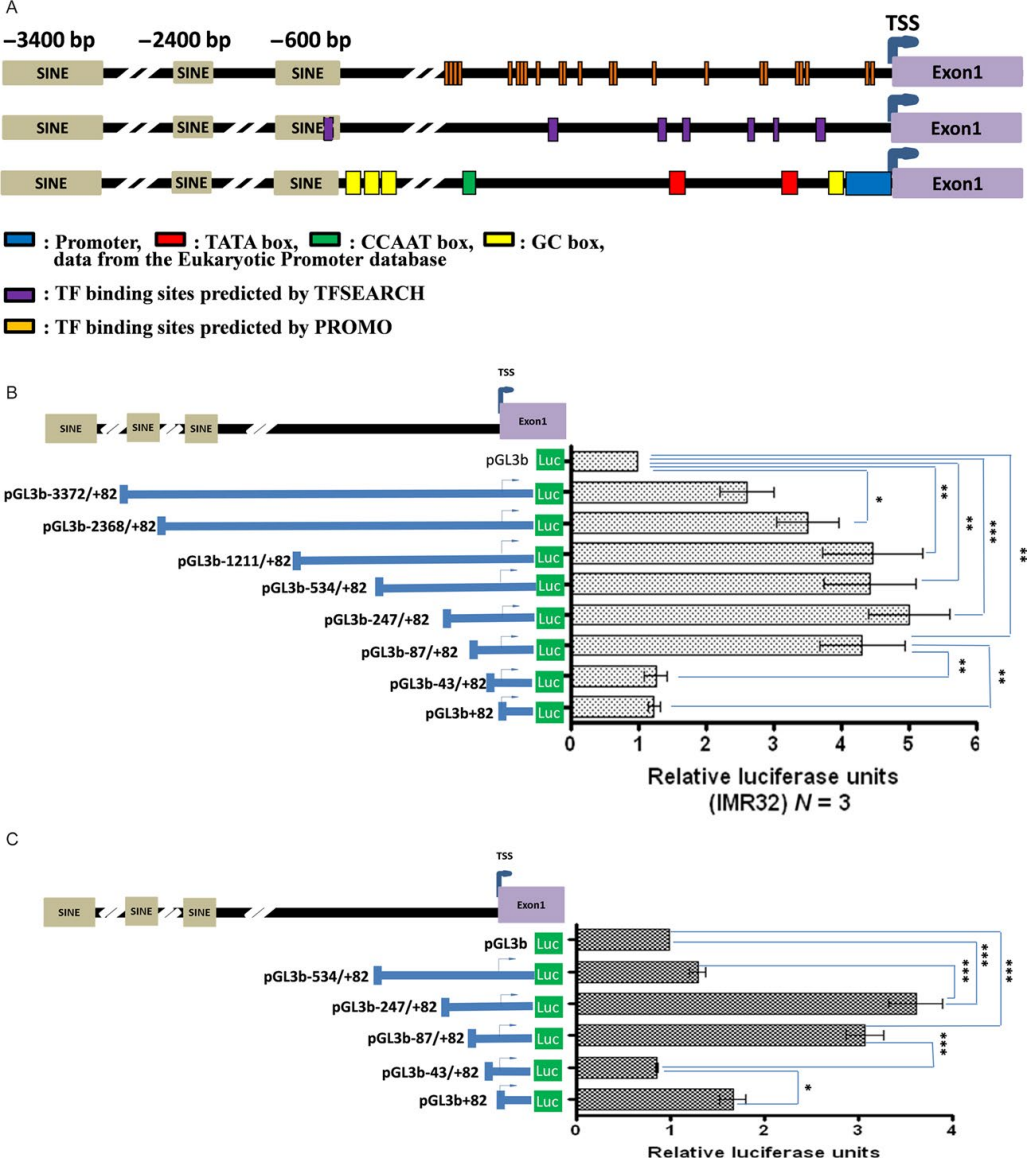
Defining the *TIAM2S* minimal core promoter region. (A) Sequences upstream from the TSS (blue arrowhead) of *TIAM2S* were used to analyze the potential promoter and TF‐binding sites. Results depicted are from the Eukaryotic Promoter database, TFSEAR, and PROMO. The presences of promoter or TF‐binding sites are indicated as boxes. Luciferase assays as representations for promoter activity were performed in (B) IMR32 and (C) HepG2 cells for constructs containing different *TIAM2S* upstream sequences. The positions of cloned sequences relative to the TSS are shown on the left panel. Measured luciferase activity relative to the empty vector (pGL3b) is indicated in the right panel. Relative luciferase activities were compared using one‐way ANOVA (*P <* *0.05, **P <* *0.01, ***P <* *0.001). Data were generated from three independent experiments.

The promoter is the modulatory DNA element that interacts with various protein factors to initiate transcription and control expression of a gene. Growing evidence supports the use of alternative promoters as a versatile mechanism to create diversity and flexibility in the regulation of gene expression 23. Because *TIAM2L* and *TIAM2S* show distinct mRNA expression patterns 1, we speculated that the two mRNA variants are generated by alternative promoters. To test this possibility, we first applied computational tools to predict the possible TF‐binding sites and potential promoter region. According to the search results from the Eukaryotic Promoter Database (EPD, http://www.epd.isb-sib.ch/), the *TIAM2S* promoter is located at −1 to −48 bp from the TSS (typically +1). In addition, two TATA boxes (−155 bp to −139 bp and −268 bp to 252 bp), one CCAAT box (−477 bp to −464 bp) and one GC box (−70 bp to −55 bp) were recorded in the EPD (Fig. 1A). However, although many TF‐binding sites were identified, no conventional promoter was identified upstream from the TSS of *TIAM2S*, even when we used two additional bioinformatics predictions (Fig. 1A).

To define the core promoter region of *TIAM2S*, we cloned approximately 3000 base pair sequences upstream from the *TIAM2S* TSS to a luciferase reporter gene system (pGL3) and further constructed serial deleted plasmids. The relative luciferase activity was measured and normalized to the cotransfected Renilla luciferase activity and further standardized to the promoterless plasmid (pGL3‐basic). The results from the deletion constructs revealed that the upstream regions of *TIAM2S* possess promoter activities in both IMR32 (Fig. 1B, *P* < 0.001) and HepG2 (Fig. 1C, *P* < 0.001) cells. Although the deletion of −87 to −43 sequences from the TSS significantly reduced luciferase activity to the basal level in both cell lines (*P *<* *0.001), removal of the predicted promoter region (i.e., pGL3B −43/+82) showed no additional decrease in the luciferase activity compared to the construct containing the −87 to −43 sequences. Therefore, the results suggested that the minimal core promoter of *TIAM2S* is located within the −87 to −43 region. The minimal core promoter region contains no conservative TATA or CCAAT box. Nevertheless, a GC box and Gata1‐recognizing site were predicted in this region. Because Gata1 is a tissue‐specific TF in erythrocytes, megakaryocytes, and eosinophils, we focused on the GC box for further examination.

### Sp1 specifically binds to the *TIAM2S* upstream core promoter region

**Figure 2:**
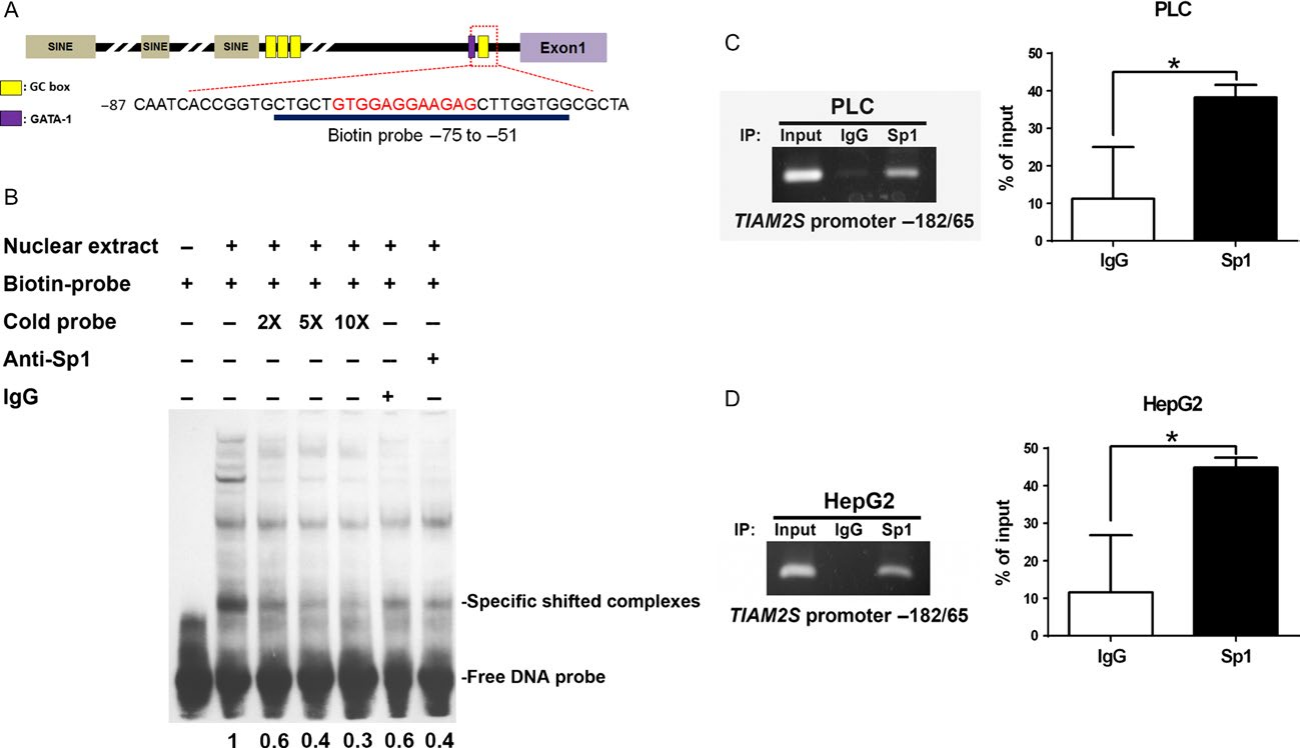
Sp1 specifically binds to the *TIAM2S* upstream core promoter region. (A) Predicted TF‐binding sites on the *TIAM2S* core promoter region. The ‐87 to ‐51 regions of the *TIAM2S* upstream sequences are shown. The underline marks biotin‐labeled sequences that were used in electrophoretic mobility shift assay (EMSA) analysis. (B) EMSAs were performed using biotin‐labeled probes and nuclear extracts isolated from HepG2 cells. From left to right, lanes 1 and 2 were probe only and with nuclear extract, respectively. For the competition assay, 2‐, 5‐, or 10‐fold of unlabeled probe was added (lanes 3‐5). IgG served as the negative control for the DNA‐protein binding assay (lane 6), and 500 ng of anti‐Sp1 antibodies were used in the binding reaction and showed specific DNA–protein interaction in the supershift assay (lane 7). ChIP assays were performed in the PLC/PRF/5 (C) and HepG2 (D) cells. The represented images indicate that the *TIAM2S* promoter is specifically bound by anti‐Sp1 antibodies (left panels, C and D). The bar charts (right panels, C and D) show the quantitative signals from Sp1 relative to the control IgG. Results were calculated from three independent experiments. *P <* *0.05, **P <* *0.01, ***P <* *0.001.

Because Sp1 is a TF that binds to the GC‐box of many promoters to regulate the expression of its target genes, we examined whether Sp1 can bind to the GC box of *TIAM2S* promoter sequences. We used biotin‐labeled oligonucleotides containing GC box sequences (−75 to −51 bases from the TSS, Fig. 2A). Results from the EMSA assay clearly illustrated that labeled probes formed different complexes with HepG2 nuclear extracts (Fig. 2B, lanes 1 and 2). When using 2X, 5X, and 10X unlabeled probes to compete with biotin‐labeled probes, our data revealed a reduction of 60%, 40%, and 30% in the band density of specific shifted complex that indicates the binding of proteins to the oligonucleotides is specific (Fig. 2B; lanes 3, 4, and 5). To confirm that the DNA‐protein complex formed by Sp1 interacts with biotin‐labeled oligonucleotides, a specific anti‐Sp1 antibody was used to compete with the binding of Sp1 to the biotin‐labeled oligonucleotides. The result showed approximately 40% reduced intensity for the complex with Sp1 specific antibody (Fig. 2B; lane 7 vs. lane 2), thus suggesting that Sp1 specifically binds to the GC‐box sequences of the *TIAM2S* promoter in vitro.

Next, we conducted ChIP experiments to confirm Sp1 chromatin occupancy on the *TIAM2S* promoter in 2 HCC cell lines. After formaldehyde cross‐linking and sonication to shear the chromatin, Sp1‐DNA complexes were immunoprecipitated by the anti‐Sp1 antibody or rabbit IgG. The precipitated DNA and input control were subjected to PCR amplification by using a set of designed primers for the GC box within the *TIAM2S* promoter (−182 to 65). Compared with the control rabbit IgG, significant 3.5‐ and 3.4‐fold enrichments were obtained for PLC/PRF/5 (Fig. 2C, *P* < 0.05) and HepG2 (Fig. 2D, *P* < 0.05), respectively. These data further support the notion that Sp1 specifically binds to the GC box of the *TIAM2S* core promoter.

### Sp1 expression, but not phosphorylation, promotes human *TIAM2S* mRNA expression

**Figure 3:**
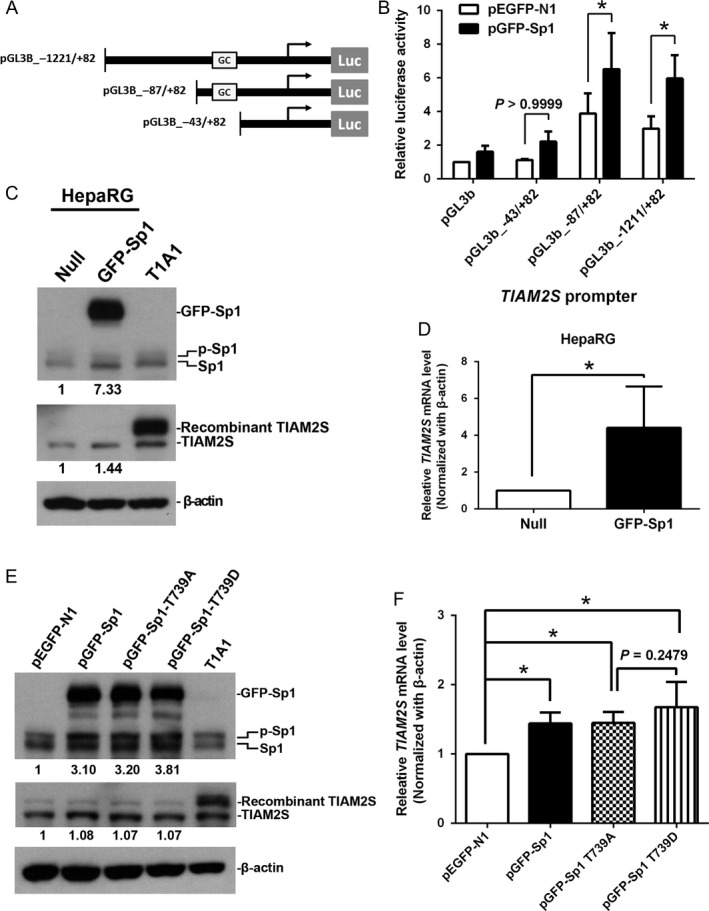
Sp1 controls *TIAM2S* transcription both in vitro and in vivo. (A) Serial *TIAM2S* deletion constructs of luciferase reporter plasmids used for transient transfection. (B) Overexpression of Sp1 increased the level of luciferase activity driven by a *TIAM2S* promoter containing a GC box. The pGL3‐b and pEGFP‐N1 served as negative controls for the promoter assay and overexpression assay, respectively. (C) Levels of endogenous Sp1 (upper panel) and *TIAM2S* (middle panel). Relative protein levels were quantified using spot‐density analysis and normalized to *β*‐actin. (D) Relative *TIAM2S* mRNA levels in HepaRG cells were measured using qRT‐PCR analysis. (E) Levels of endogenous Sp1 (upper panel, left) and *TIAM2S* (middle panel, left) in cells transfected with various Sp1 constructs in PLC/PRF/5 cells. The potential Sp1 phosphorylated threonine sites were mutated to alanine (T739A, constructive inactivated form) and aspartate (T739D, constructive activated form) as indicated. pEGFP‐N1 served as a negative control. Relative protein levels of Sp1 and *TIAM2S* were quantified using spot‐density analysis and normalized to *β*‐actin. The *TIAM2S* stable clone T1A1 was used as a positive control for *TIAM2S* expression. (F) Relative *TIAM2S* mRNA levels in various *SP1*‐mutant expressing cells were measured and compared. “p‐Sp1” represents phosphorylated Sp1. *P <* *0.05.

To elucidate whether Sp1 controls *TIAM2S* mRNA expression, we cotransfected different *TIAM2S* promoter constructs (Fig. 3A) with an Sp1‐expressing plasmid in the promoter activity assays. The results showed that reporter activities were 3.5‐ (pGL3b_−87/+82; *P *<* *0.05) and 2.7‐fold (pGL3b_−1211/+82; *P *<* *0.05) higher in the constructs containing a *TIAM2S* core promoter than in the constructs that contained no core promoter sequences (pGL3b_−43/+82) (Fig. 3B). When exposed to Sp1 overexpression, promoter activities further increased 1.7‐ (pGL3b_−87/+82; *P *<* *0.001) and 2‐fold (pGL3b_−1211/+82; *P *<* *0.01), respectively (Fig. 3B). The data suggest that Sp1 recognizes the *TIAM2S* GC box sequences and activates *TIAM2S* promoter activity. Therefore, the upstream GC box of *TIAM2S* is essential for Sp1‐mediated *TIAM2S* promoter activity.

To elucidate whether *TIAM2S* expression is mediated by Sp1, we overexpressed human Sp1 and measured *TIAM2S* expression in the HepaRG cell line, which is a terminally differentiated hepatic cell line that retains many characteristics of primary human hepatocytes cells (Fig. 3C). Sp1 overexpression was assayed using a Western blot and displayed an approximately sevenfold elevation in HepaRG cells (Fig. 3C, top panel). Furthermore, overexpressing Sp1 led to an approximately 1.5‐fold increment on *TIAM2S* protein expression (Fig. 3C, second panel) that is comparable to the induction rates of Sp1‐induced luciferase activities (Fig. 3B). Nevertheless, *TIAM2S* mRNA expression increased fourfold in HepaRG cells (Fig. 3D).

Previous studies have shown that phosphorylation at Thr739 is critical for Sp1 transcriptional activity 24. To characterize the regulatory mechanism underlying Sp1‐mediated *TIAM2S* expression, we overexpressed two Sp1 Thr739 mutant constructs in which the potential Sp1 phosphorylated threonine site was mutated to alanine (T739A, constructive inactivated form) or aspartate (T739D, constructive activated form) in the PLC/PRF/5 cells and examined the expressions of endogenous *TIAM2S* mRNA and protein. Similar to the results presented in Figure 3C, all three Sp1 overexpression constructs successfully elevated Sp1 expression >threefold compared with the empty vector control (Fig. 3E; top panel). Although increased Sp1 significantly promoted *TIAM2S* mRNA expression 1.4‐, 1.4‐, and 1.5‐fold in cells transfected with the wild‐type (pGFP‐Sp1, *P *<* *0.05), constructive inactivated (pGFP‐Sp1‐T739A, *P *<* *0.05), and constructive activated (pGFP‐Sp1‐T739D, *P *<* *0.05) constructs (Fig. 3F), the elevated levels among them were similar (Fig. 3F; *P *=* *0.60), with no difference between cells transfected with constructively inactivated and activated forms (Fig. 3F; *P *=* *0.25).

These results indicated that phosphorylation at the Sp1 Thr739 is not required to activate the *TIAM2S* promoter. Nevertheless, the additional recombinant Sp1 introduced by constructs still slightly increased *TIAM2S* protein amounts regardless of the abundant expressions of endogenous Sp1 and *TIAM2S* in the PLC/PRF/5 cells (Fig. 3E; middle panel). Collectively, results from these experiments suggested that the upstream GC box in the *TIAM2S* promoter is necessary and sufficient to interact with Sp1 to fully activate *TIAM2S* mRNA expression. In addition, these data also demonstrate that the presence of the Sp1 protein is adequate to activate the *TIAM2S* promoter for transcription. The phosphorylation of Sp1 is not required and also contributed no additional effect on *TIAM2S* mRNA expression.

### Sp1 positively regulates human *TIAM2S* mRNA expression in HCC cells

**Figure 4:**
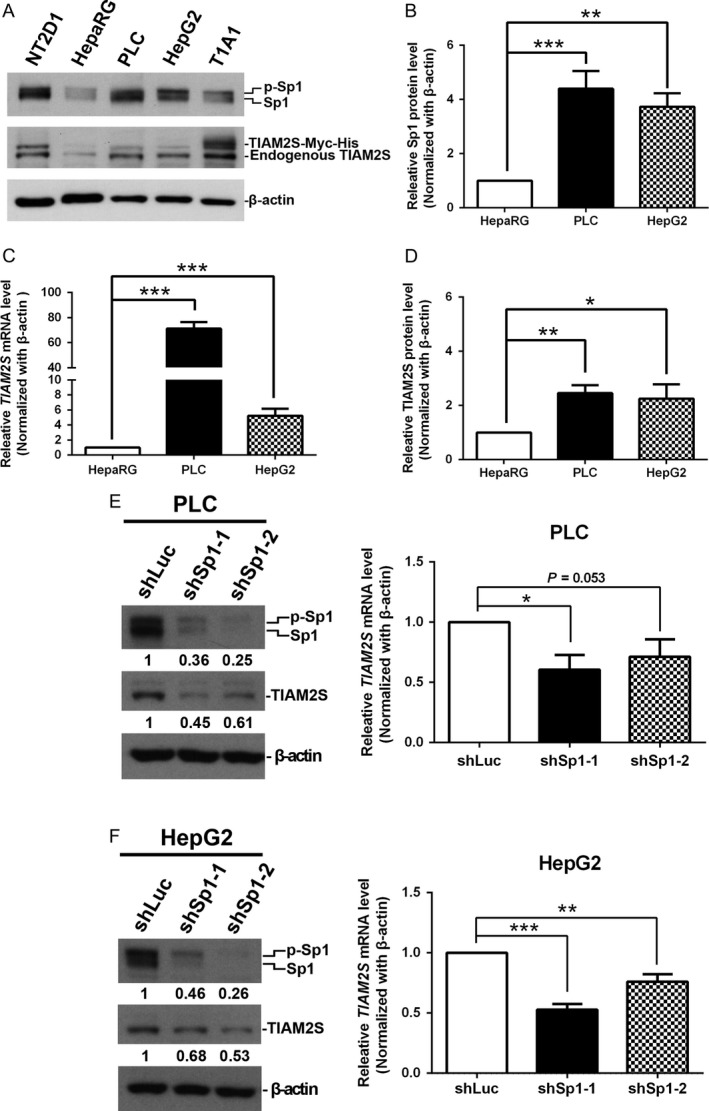
Sp1 positively regulates human *TIAM2S* mRNA expression in HCC cells (A) Endogenous levels of Sp1 and *TIAM2S* in different HCC cell lines were measured using Western blotting. The bar chart shows the quantified expressions of Sp1 protein (B), *TIAM2S* mRNA (C), and *TIAM2S* protein (D) between two HCC cell lines and one normal liver cell line (i.e., HepaRG). Both NT2D1 and T1A1 were positive controls. *β*‐Actin was a loading control. (C) and (D) The expression levels of both the *TIAM2S* mRNA (C) and protein (D) were increased by Sp1 overexpression in PLC/PRF/5 and HepG2 cells. Western blots showed that the endogenous levels of Sp1 were knocked down by anti‐Sp1 shRNA lentivirus (10 MOI for 48 h) in PLC/PRF/5 (E) and HepG2 (F) cells. Therefore, the results from qRT‐PCR showed that the *TIAM2S* mRNA was significantly reduced in Sp1‐knockdown PLC/PRF/5 (E, left) and HepG2 (F, left) cells. *P <* *0.05, **P <* *0.01, ***P <* *0.001.

To determine whether Sp1 plays any role in the ectopic expression of *TIAM2S* in HCC cells, we first examined the expressions of Sp1 protein in various HCC cell lines (Fig. 4A). Compared with normal liver cells (i.e., HepaRG), Sp1 expression was significantly increased 4.4‐ and 3.7‐fold (Fig. 4B) in PLC/PRF/5 (*P *<* *0.001) and HepG2 (*P *=* *0.001) liver cancer cells. Moreover, we found that *TIAM2S* mRNA expressions were also 71.2 and 5.2 times higher in PLC/PRF/5 (*P *<* *0.001) and HepG2 (*P *<* *0.001), respectively, compared with the HepaRG cells (Fig. 4C). Although the magnitude is not as large as the magnitude of mRNA, 2.5‐ and 2.3‐fold increases in *TIAM2S* protein expressions in PLC/PRF/5 (*P *<* *0.01) and HepG2 (*P *<* *0.05) were also detected (Fig. 4D).

To define the effect of Sp1 on *TIAM2S* ectopic expression, we knocked down endogenous Sp1 protein expression by stably infecting cells with Sp1‐specific shRNA in PLC/PRF/5 (Fig. 4E) and HepG2 (Fig. 4F) cells. Compared with the shLuc control, Sp1‐specific shRNAs successfully knocked down Sp1 expression to between 11% and 37% in PLC/PRF/5 cells (Fig. 4E, the upper left panel). Consequently, a significant reduction (*P *<* *0.05) of *TIAM2S* mRNA expression was observed (Fig. 4E, right), which led to a drop of *TIAM2S* protein to 45–61% (Fig. 4E, left and middle panels). Similar results were obtained for HepG2 cells (Fig. 4F). The knockdown of the Sp1 protein (ranging from 18% to 52%; Fig. 4F, left and upper panel) significantly decreased endogenous *TIAM2S* mRNA expression (*P *<* *0.001 and 0.01 for shSp1‐1 and shSp1‐2, respectively; Fig. 4F, right) and resulted in a ~40% decline in the *TIAM2S* protein. Together with the results of Sp1 overexpression (Fig. 3C and 3D), these data demonstrate that Sp1 controls the ectopic expression of human *TIAM2S* mRNA in HCC cells.

### Elevated Sp1 expression promotes *TIAM2S* aberrant expression in HCC cells

**Figure 5:**
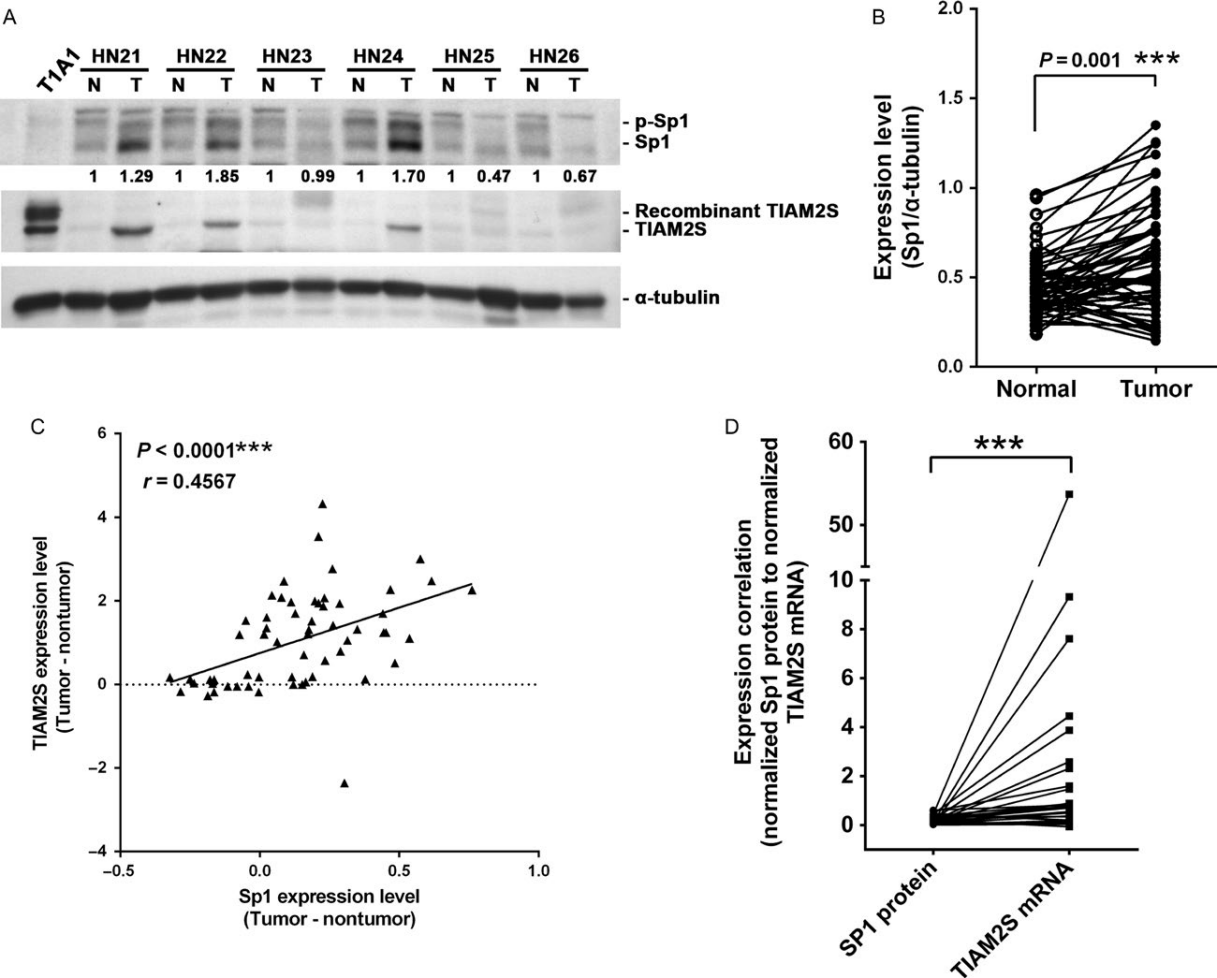
Sp1 controls *TIAM2S* ectopic expression in HCC cells. (A) Expression levels of endogenous Sp1 (upper panel) and TIAM2S (middle panel) detected using Western blotting from six paired HCCs. TIAM2S stable clone T1A1 and *α*‐tubulin were used as positive and loading controls, respectively. (B) Relative Sp1 expression levels in tumor and matched nontumor cells from 60 paired HCCs were normalized to *α*‐tubulin and plotted in pairs. (C) Correlation between elevated expressions of Sp1 and TIAM2S from 60 paired HCCs were plotted and showed. (D) Using qRT‐PCR in 26 paired HCC cases, the results showed substantial correlation between the Sp1 protein level and *TIAM2S* mRNA amount. ***P <* *0.001.

To confirm the Sp1‐mediated *TIAM2S* expression in a clinical setting, we examined the expression levels of the Sp1 protein in 60 paired HCC samples (Fig. 5A and Table S2, Fig. 2A–J). Compared with the matched nontumor part, 42 (70%) paired HCCs showed an increase (from 1.1‐ to 3.6‐fold) in Sp1 protein expression in the tumor cells (Fig. 5B, *P* < 0.001). Combined with the expression levels of *TIAM2S* measured in our previous work 1, we found a highly significant correlation between Sp1 and *TIAM2S* expressions in these HCC samples (Fig. 5C; *r* = 0.4567, *P* < 0.0001). To elucidate the transcriptional role of Sp1 protein in the ectopic expression of TIAM2S mRNA, we quantified TIAM2S mRNA in these HCC samples. Using 26 paired cases with mRNA available, the results from quantitative reverse transcription PCR (qRT‐PCR) showed a substantial correlation between the Sp1 protein level and *TIAM2S* mRNA amount (Fig. 5D; *P* < 0.0001). These results indicated that the ectopic expression of *TIAM2S* in HCC cells is mediated, at least partially, by the elevated expression of Sp1 in these cancer cells.

## Discussion

The expression of tissue‐specific genes out of their normal and physiological setting has gained much attention recently 25, 26. The ectopic activation of these cell‐ and tissue‐specific factors in cancers represents a promising source of cancer biomarkers and targets for new therapeutic approaches 27. *TIAM2S* was undetectable in normal liver cells but highly expressed in a great majority (86%) of HCCs, which suggests the potential of *TIAM2S* as a target for antitumor therapy and cancer intervention 1. Although our previous study showed solid evidence that the induction of endogenous *TIAM2S* in the liver promotes EMT and results in the proliferation of and invasion by liver cancer cells, the reason for and mechanism of *TIAM2S* activation and expression in liver cancer cells remain largely unknown. Because the *TIAM2L* protein was undetected in our previous works, we suspect that *TIAM2L* and *TIAM2S* are under distinct transcriptional controls for tissue‐specific mRNA expression. In addition, *TIAM2S* has a unique first exon that is embedded within the intron 14 of the *TIAM2L*. Thus it is suggested that *TIAM2S and TIAM2L* may be generated by different promoters. In this study, we showed that *TIAM2S* expression is controlled by a TATA‐less promoter located at −43 to −87 bps upstream from the TSS of *TIAM2S* gene. The identification of the core promoter of *TIAM2S* explains the differential expression patterns of *TIAM2S* and *TIAM2L* in various tissues observed previously 1.

Although the in silico analysis revealed that *TIAM2S* lacks a conventional promoter such as a TATA or CCAT box, a single Sp1‐binding site within the GC‐rich core promoter region of *TIAM2S* was identified. Studies have demonstrated that Sp1 is a common TF that controls the transcriptional activity of genes implicated in most cellular processes 6, 7, 8. In addition, the Sp1 protein plays a critical role in the regulation of tissue‐specific and cancer‐enriched genes by binding directly onto the GC/GT boxes of these genes 28. We thus speculated that Sp1 may contribute to the ectopic expression of *TIAM2S* in HCC cells. As expected, our results indicated that Sp1 specifically binds to the GC‐box of the *TIAM2S* core promoter and controls *TIAM2S* mRNA expression.

Previous studies have demonstrated the pleiotropic roles of Sp1; it can either activate 29 or repress the expression of the target genes. For example, Sp1 positively regulates *MTA2* and midkine (MDK) expressions in gastric cancer tissues 30 and glioma cells 29, respectively. Additionally, Sp1 has demonstrated the ability to inhibit DsbA‐L gene transcription in a mouse model. The Sp1‐mediated inhibition of DsbA‐L gene expression may be responsible for obesity‐induced adiponectin downregulation and insulin resistance 31. Moreover, the binding of Sp1 to the PTEN core promoter inhibits PTEN expression and results in increased cancer cell migration and invasion 32. Our study demonstrated that Sp1 knockdown reduced the expression of *TIAM2S* in both HepG2 and PLC/PRF/5 cells, whereas Sp1 overexpression increased *TIAM2S* expression in HepaRG cells. Overall, these results suggest that Sp1 binds to the GC box sequences residing within the *TIAM2S* core promoter region and positively regulates *TIAM2S* transcription in HCC cells.

The expression of *TIAM2S* promotes proliferation and invasion in liver 1 and lung 2 cancer cells. It was unclear previously how the expression of *TIAM2S* was undetected in the normal cells but activated in the cancer cells. In this study, we identified Sp1 as the TF to activate *TIAM2S* expression in these cancer cells. Although the phosphorylation of Sp1 has been shown to modulate transcriptional activity and affect the gene expression and biological functions of Sp1 24, 33, we found that the T739 phosphorylation of Sp1 was not necessary to trigger *TIAM2S* mRNA transcription. Therefore, the data suggested that the mechanism of Sp1 in controlling *TIAM2S* gene expression is at the expression level of Sp1 in these cells. This notion is supported by our previous report, which indicated that the sumoylation of Sp1 is attenuated during tumorigenesis to increase Sp1 stability and results in the accumulation of the Sp1 protein, as observed in various tumors 11. Although the presence of the Sp1 protein in HCC cells explains the majority of *TIAM2S* expression in these cancer cells, a few cases where *TIAM2S* expression under low or no expression of Sp1 indicate the possibility of other regulatory mechanisms being involved in controlling *TIAM2S* expression. For example, Sp1 was found to affect the chromatin accessibility of *CD151* and P2X7 receptor promoters in liver cancer cells 34 and neuroblastoma cells 35, respectively. These results therefore suggest that chromatin remodeling may be separately or jointly involved in Sp1‐mediated *TIAM2S* expression in cancer cells. The effect of epigenetic regulation on *TIAM2S* ectopic expression should be investigated. Nevertheless, this study provides the first step to reveal the detailed mechanism of controls of *TIAM2S* expression in HCC cells. This information may provide an alternative target for clinical therapy in controlling liver cancers.

## Conflict of Interest

None declared.

## Supporting information


**Table S1.** Primer sequences used in this study.
**Table S2.** Detailed information of all HCC cases.
**Figure S1.** Sp1 overexpression increased the expression level of *TIAM2S* mRNA in HCC cells.
**Figure S2.** Strong positive correlation between the expressions of Sp1 and TISM2S in HCC patients.
