# Supplementary material for: Sp1‐mediated ectopic expression of T‐cell lymphoma invasion and metastasis 2 in hepatocellular carcinoma
Source: Cancer Med. 2016 Jan 14;5(3):465–77. doi: 10.1002/cam4.611 (PMC4799941; doi:10.1002/cam4.611)
Supplement: Supplementary file 1 — Table S1. Primer sequences used in this study. Table S2. Detailed information of all HCC cases. Figure S1. Sp1 overexpression increased the expression level of TIAM2S mRNA in HCC cells. Figure S2. Strong positive correlation between the expressions of Sp1 and TISM2S in HCC patients. [file CAM4-5-465-s001.docx]

**Sp1-mediated ectopic expression of T-cell lymphoma Invasion and Metastasis 2 (TIAM2) in hepatocellular carcinoma**

Wei-Hsuan Yen^1^, Wu-Sian Ke^1^, Jan-Jong Hung^2^, Tsung-Ming Chen^3^, Jia-Shing Chen^1^, H. Sunny Sun^1^

^1^Institute of Molecular Medicine, College of Medicine, National Cheng Kung University, Tainan 70101, Taiwan.

^2^Institute of Bioinformatics and Biosignal Transduction; College of Bioscience and Biotechnology, National Cheng Kung University, Tainan 70101, Taiwan.

^3^ Department of Physiology, College of Medicine, National Cheng Kung University, Tainan 70101, Taiwan.

**Running title: Sp1 controls TIAM2S expression in HCC**

Corresponding author:

H. Sunny Sun, PhD

Institute of Molecular Medicine, College of Medicine, National Cheng Kung University

1 University road

Tainan 70101, Taiwan

Phone: 886-6-235-3535 ext. 3648

FAX: 886-6-209-5845

Email: hssun@mail.ncku.edu.tw

**Supplementary Data**

**Table S1. Primer sequences used in this study**

| **ID** | **Sequence from 5' to 3'^*^** | **Description** |
| --- | --- | --- |
| **TIAM2S_-3372_F** | GGTACCACTCTGGVCTGTGGGACTGG | pGL3b_-3372/+82 |
| **TIAM2S_-2368_F** | AGAGGTACCGCCTGGTTATGTCCCAGGATGCAAGTTATT | pGL3b_-2368/+82 |
| **TIAM2S_+82_R** | CTCAAGCTTGGAGAACGGATCACATCTAAGTCGGCTGTT | pGL3b_-2368/+82 |
| **TIAM2S_-1211_F** | AGAGGTACCCGTGTTATATCTAAAGCAATCCTCTTCTGG | pGL3b_-1211/+82 |
| **TIAM2S_-534_F** | AGAGGTACCAGCACAGCTGTACCCAGGGCTTTTG | pGL3b_-534/+82 |
| **TIAM2S_-247_F** | AGT GGT ACC AGA CAT GCT GCC TGC ACT CCA CCT | pGL3b_-247/+82 |
| **TIAM2S_-87_F** | AGAGGTACCCAATCACCGGTGCTGCTGTGGA | pGL3b_-87/+82 |
| **TIAM2S_-43_F** | GGCGGTACCACGTCCTTGTGAATGTGACTTGGAAGA | pGL3b_-43/+82 |
| **TIAM2S_+1_F** | CCCGGTACCTTCATTGCCTGGGGATGAGAGGG | pGL3b_-43/+82 |
| **TIAM2S_+82 R** | CTCACGCGTGGAGAACGGATCACATCTAAGTCGG | pGL3b_+82 |

^*^ Underlines mark the restriction enzyme cutting site. All sequences are with KpnI cutting site except the TIAM2S_+82_R and TIAM2S_+82 R were designed to be cut with *Hind* Ⅲ and *Mlu*Ⅰ, respectively.

Table S2. Detailed information of all HCC cases

| No. | Tumor  type^A^ | Pathology stage^B^ | TIAM2S | | | Sp1 |
| --- | --- | --- | --- | --- | --- | --- |
|  |  |  | mRNA | Protein^C^  N/T | | Protein^C^  N/T |
| HN1 | 2 | II | NA | | 0.129/2.071 | 0.316/0.526 |
| HN2 | 1 | II | NA | | 0.184/0.000 | 0.238/0.234 |
| HN3 | 2 | II | NA | | 0.047/2.116 | 0.284/0.516 |
| HN4 | 1 | II | NA | | 0.057/2.531 | 0.317/0.933 |
| HN5 | 1 | I | NA | | 0.000/0.057 | 0.342/0.507 |
| HN6 | 2 | II | NA | | 0.000/1.019 | 0.340/0.402 |
| HN7 | 2 | IIIA | NA | | 0.000/2.132 | 0.347/0.391 |
| HN8 | 1 | I | NA | | 0.000/0.510 | 0.203/0.687 |
| HN9 | 1 | I | NA | | 0.000 /0.235 | 0.332/0.288 |
| HN10 | 1 | II | NA | | 0.000/0.000 | 0.608/0.76 |
| HN11 | 1 | II | NA | | 0.052/0.228 | 0.521/0.636 |
| HN12 | 2 | IIIA | NA | | 0.026/1.376 | 0.446/0.470 |
| HN13 | 2 | IIIB | NA | | 0.000/1.698 | 0.425/0.865 |
| HN14 | 2 | IIIA | NA | | 0.000/1.199 | 0.468/0.483 |
| HN15 | 2 | IIIA | NA | | 0.156/1.207 | 0.308/0.621 |
| HN16 | 1 | IIIA | NA | | 0.292/0.119 | 0.488/0.204 |
| HN17 | 2 | IIIA | NA | | 0.000/1.192 | 0.528/0.455 |
| HN18 | 1 | IIIA | NA | | 0.059/2.050 | 0.552/0.749 |
| HN19 | 1 | I | NA | | 0.000/0.020 | 0.343/0.174 |
| HN20 | 1 | I | NA | | 0.190/1.289 | 0.447/0.984 |
| HN21 | 2 | IIIA | NA | | 0.169/1.870 | 0.441/0.568 |
| HN22 | 1 | I | NA | | 0.078/1.396 | 0.410/0.759 |
| HN23 | 2 | II | NA | | 0.217/0.394 | 0.494/0.491 |
| HN24 | 1 | I | NA | | 0.046/1.289 | 0.634/1.078 |
| HN25 | 1 | I | NA | | 0.054/0.181 | 0.472/0.221 |
| HN26 | 2 | IIIA | NA | | 0.142/0.253 | 0.537/0.358 |
| HN27 | 1 | IIIA | NA | | 0.000/1.209 | 0.229/0.401 |
| HN28 | 1 | I | NA | | 0.000/0.795 | 0.469/0.758 |
| HN29 | 1 | I | NA | | 0.000/0.573 | 0.852/1.086 |
| HN30 | 2 | II | NA | | 0.000/2.258 | 0.495/1.256 |
| HN31 | 1 | I | NA | | 0.000/4.325 | 0.961/1.186 |
| HN32 | 1 | I | NA | | 4.054/11.011 | 1.616/3.288 |
| HN33 | 1 | II | NA | | 0.517/2.045 | 0.371/0.320 |
| HN34 | 1 | II | NA | | 0.578/2.090 | 0.315/0.501 |
| HN35 | 2 | IIIB | NA | | 0.561/0.289 | 0.384/0.198 |
| HN36 | 2 | IIIA | NA | | 0.268/0.085 | 0.523/0.359 |
| HN37 | 1 | IIIB | NA | | 0.655/3.659 | 0.775/1.351 |
| HN38 | 1 | IIIB | NA | | 3.834/1.473 | 0.944/1.247 |
| HN39 | 2 | II | NA | | 0.079/2.011 | 0.336/0.622 |
| HN40 | 1 | I | NA | | 0.196/3.732 | 0.390/0.601 |
| HN41 | 1 | IIIA | NA | | 0.046/0.000 | 0.260/0.219 |
| HN42 | 1 | II | NA | | 0.041/2.513 | 0.273/0.359 |
| HN43 | 1 | I | NA | | 0.132/2.899 | 0.433/0.694 |
| HN44 | 1 | I | NA | | 0.314/0.440 | 0.592/0.970 |
| HN45 | 1 | I | NA | | 0.074/1.382 | 0.731/0.906 |
| HN46 | 1 | I | NA | | 0.048/0.000 | 0.365/0.250 |
| HN47 | 1 | II | NA | | 0.545/2.513 | 0.508/0.620 |
| HN48 | 1 | I | NA | | 0.045/0.228 | 0.573/0.762 |
| HN49 | 1 | I | NA | | 0.864/2.282 | 0.394/0.656 |
| HN50 | 2 | II | NA | | 0.201/1.807 | 0.393/0.417 |
| HN51 | 1 | I | NA | | 0.037/0.066 | 0.425/0.189 |
| HN52 | 1 | II | NA | | 0.000/0.000 | 0.309/0.429 |
| HN53 | 1 | I | NA | | 0.048/0.000 | 0.430/0.348 |
| HN54 | 1 | I | NA | | 0.000/0.170 | 0.682/0.360 |
| HN55 | 1 | II | NA | | 0.123/1.367 | 0.335/0.787 |
| HN56 | 2 | IIIA | NA | | 0.097/1.971 | 0.621/0.848 |
| HN57 | 1 | IIIA | NA | | 0.113/0.231 | 0.310/0.147 |
| HN58 | 1 | IIIA | NA | | 0.216/2.299 | 0.415/0.492 |
| HN59 | 1 | I | NA | | 0.000/2.267 | 0.183/0.651 |
| HN60 | 2 | II | NA | | 0.049/0.754 | 0.341/0.498 |

^A^ 1 and 2 are for solitary and multiple type of tumor.

^B^: The classification of Pathology stages were according to AJCC, UICC, and CUPI.

^C^: N and T indicated normal and tumor respectively. Expression of TIAM1 and TIAM2S protein were presented by the protein amount in paired HCCs as measured by spot density and normalized with α-tubulun.

**Figure S1**

***a*. *b*.**


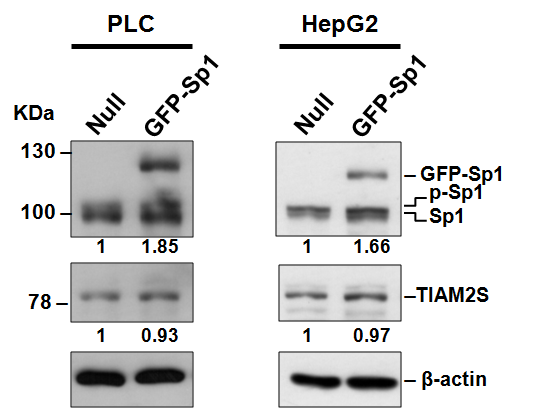

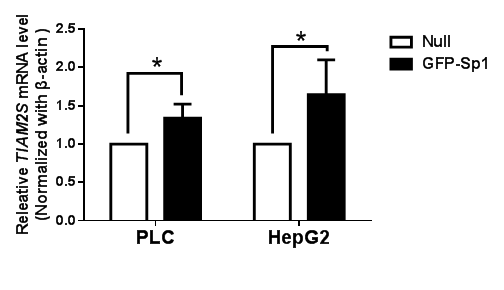


**Figure S1. Sp1 overexpression increased the expression level of *TIAM2S* mRNA in HCC cells.** (a) Western blotting showed that the Sp1 was overexpressed in both PLC and HepG2 cells, while the endogenous TIAM2S protein were unaffected in these 2 cell lines. (b). RT-qPCR showed that the *TIAM2S* mRNA was increased in Sp1-overexpressed cells.

**Figure S2**

(*a*)


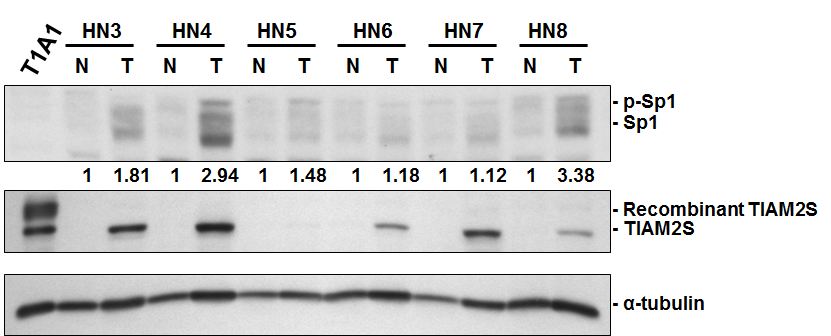


(*b*)


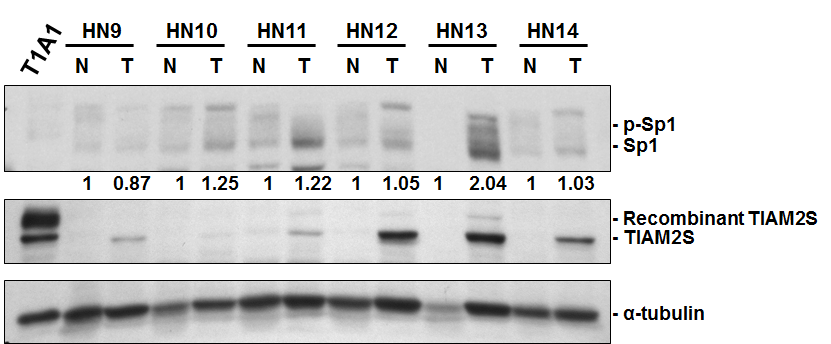


(*c*)


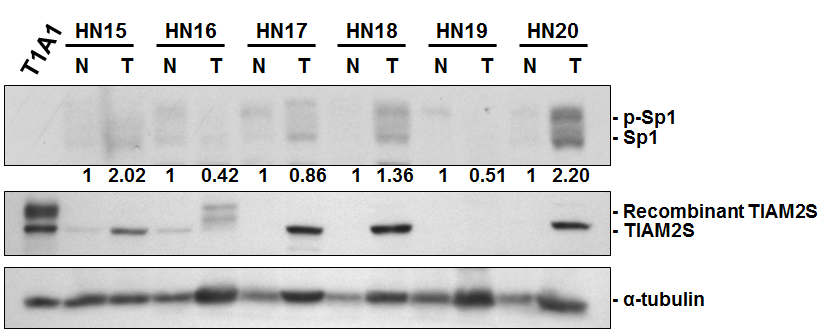


(*d*)


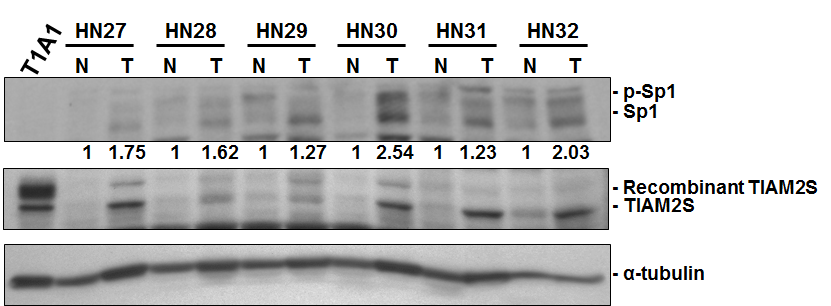


(*e*)


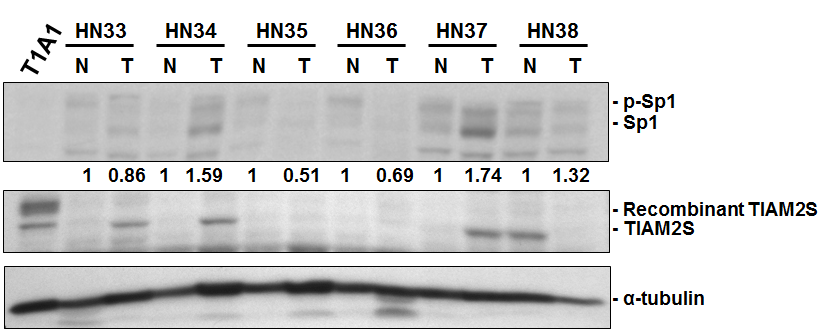


(*g*)


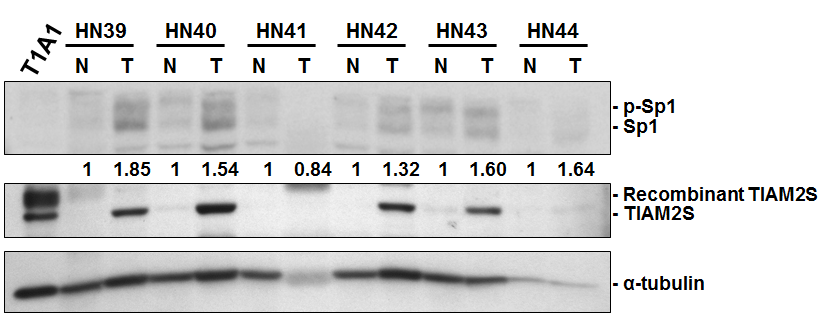


(*h*)


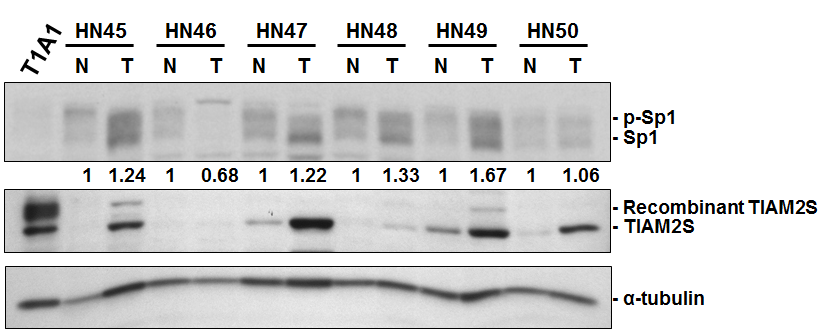


(*i*)


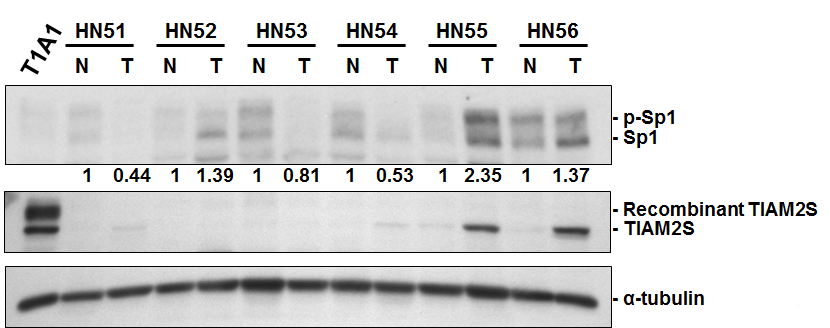


(*j*)


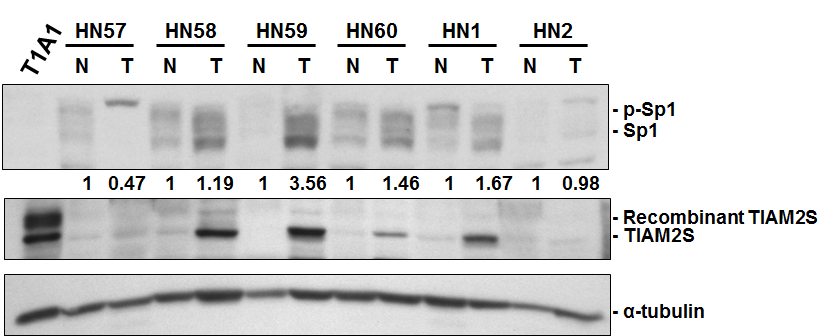


**Figure S2. Strong positive correlation between the expressions of Sp1 and TISM2S in HCC patients.**

(*a-j*) Represented images showed the expression levels of endogenous Sp1 (upper panel) and TIAM2S (middle panel) detected by Western blotting from 54 paired HCCs. TIAM2S stable clone T1A1 and α-tubulin were used as positive control and loading controls, respectively.
